# Paget’s disease of the breast: clinical, imaging and pathologic findings: a review of 16 patients

**DOI:** 10.2349/biij.7.2.e16

**Published:** 2011-04-01

**Authors:** M Muttarak, B Siriya, P Kongmebhol, B Chaiwun, N Sukhamwang

**Affiliations:** 1 Department of Radiology, Faculty of Medicine, Chiang Mai University, Chiang Mai, Thailand; 2 Department of Pathology, Faculty of Medicine, Chiang Mai University, Chiang Mai, Thailand

**Keywords:** Coronary artery disease, single photon emission computed tomography, positron emission tomography, computed tomography, diagnostic value

## Abstract

**Objectives:**

To determine the clinical, imaging and pathological findings of Paget’s disease of the breast.

**Materials and methods:**

Approval by Institutional Review Board was granted and informed consent was waived. Retrospective review of the pathological diagnosis of 2,361 women with breast carcinoma between January 2004 and April 2010 revealed 27 patients with Paget’s disease of the breast. The clinical, mammographic and ultrasonographic images were retrospectively reviewed.

**Results:**

The prevalence of Paget’s disease of the breast was 1.14% of all breast carcinoma at this institution. Of the 27 patients with Paget’s disease, only 16 had imaging studies and this group constituted the basis of this study. All 16 patients were women, with ages ranging from 36–68 years (mean age 50.31 years). Eleven patients presented with clinical findings suggestive of Paget’s disease of the breast. Seven of these 11 patients also had associated palpable mass(es). Four patients presented with a palpable mass alone and one presented with bloody nipple discharge alone. Mammography was performed in all 16 patients and ultrasonography (US) in 15 patients. Of the 16 mammographic studies, two were negative. Of the 15 US studies, three were negative. Of these three negative US studies, two also had negative mammography and one had pleomorphic microcalcifications on mammogram. US was helpful in detecting multifocality in two patients. Mammography was 100% positive in patients who presented with palpable breast mass(es) and bloody nipple discharge, but 50% positive in patients who had clinically suggestive Paget’s disease alone. Almost all patients (15/16) had underlying breast malignancies. Seven patients had multifocality or multicentricity. Modified radical mastectomy was performed in 13 patients, simple mastectomy in two, and wide local excision in one patient. Pathological findings were ductal carcinoma in situ (DCIS) (*n* = 3), invasive ductal carcinoma (IDC) (*n* = 10), metaplastic carcinoma (*n* = 1), invasive lobular carcinoma (ILC) (*n* = 1), and only Paget’s disease of the nipple without underlying breast carcinoma (*n* = 1).

**Conclusion:**

Patients with Paget’s disease of the breast have a high incidence of an underlying breast carcinoma. Most of the patients in this study presented late and were more likely to have positive mammograms. Mammography should be performed to identify the underlying breast carcinoma. Those who have only nipple areolar changes and no palpable mass have less positive mammography and less invasive carcinoma.

## INTRODUCTION

Paget’s disease of the breast is rare and was first described by Sir James Paget in 1874 [1]. The disease is characterised by the involvement of the epidermis by malignant cells called Paget cells. These malignant cells are large with abundant clear or lightly staining cytoplasm and nuclei with prominent nucleoli. Clinically, patients often present with changes in the nipple and areolar which include itching, erythema, scaly or flaky skin, bloody nipple discharge, nipple erosion or ulceration, and nipple retraction [2–5]. Although diagnosis of Paget’s disease is usually made on the basis of clinical findings, delayed correct diagnosis by suggesting a benign diagnosis of eczema may occur, resulting in treatment with topical steroids. On the other hand, some patients with Paget’s disease of the breast may not have clinical symptoms but are incidentally discovered at screening mammography [2]. Therefore, it is important for radiologists to be aware of this disease because Paget’s disease of the breast is nearly always a sign of underlying breast malignancy. However, the imaging findings of Paget’s disease of the breast have rarely been described [2–6]. Familiarity of the imaging features of Paget’s disease of the breast will help in early diagnosis and proper management. The authors reviewed the clinical presentation, imaging, and pathology of Paget’s disease of the breast at their institution.

## MATERIALS AND METHODS

The study was approved by the Institutional Review Board and informed consent was waived. The authors retrospectively reviewed pathological diagnosis of 2,361 breast carcinomas between January 2004 and April 2010. There were 27 patients with pathological diagnosis of Paget’s disease of the breast. The clinical records, including patient’s age, clinical presentation, and type of surgery, were obtained. Mammography was performed using dedicated film-screen equipment (Siemens Mammomat 3000 Nova, Germany) from 2004–2007 and the Fuji Computed Radiography System (Fuji Corporation, Tokyo, Japan) from 2008-present. Images were obtained in two standard views (mediolateral oblique (MLO) and craniocaudal (CC)), with additional views as deemed necessary. US was performed using a variety of commercially available 5–14 MHz linear array transducers (HDI 5000, Advanced Technology Laboratories, Bothell, WA, USA, Siemens, Sequoia, Acuson, CA, USA, and Toshiba Aplio XG, Japan).

Mammographic images were retrospectively reevaluated by two breast radiologists with knowledge of the pathologic report to determine the presence of focal mass, microcalcifications, architectural distortion, asymmetrical density, axillary lymphadenopathy and associated features such as skin thickening, nipple retraction, and breast edema. US images were assessed for the presence of mass and microcalcifications. Agreement on the imaging findings was by consensus. The mammographic and US findings were determined according to the American College of Radiology Breast Imaging Reporting and Data System (BI-RADS) lexicon [7]. Pathological review of all lesions were determined by two pathologists.

## RESULTS

**Figure 1:**
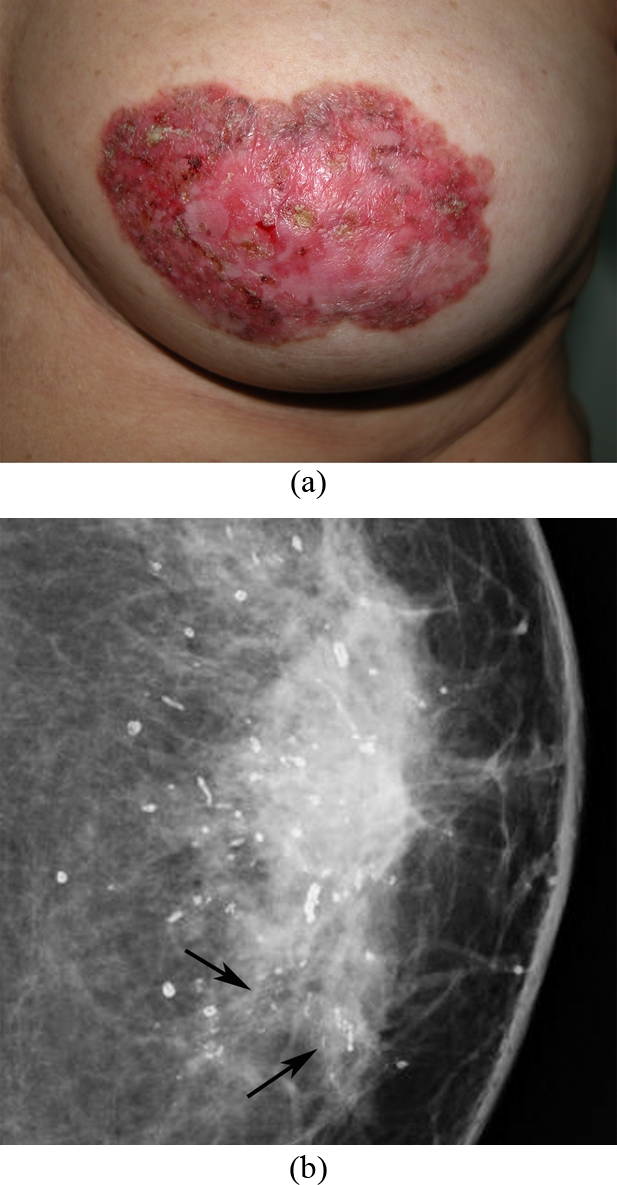
A 56-year-old woman, presented with left nipple areolar changes. (a) Photograph of the left breast shows skin thickening, redness, erythema, erosion of the nipple, and scaling around the nipple areola area. (b) Left CC mammogram shows scattered rod-like calcifications and groups of pleomorphic, fine linear microcalcifications in the inner quadrant (arrows). Simple mastectomy revealed DCIS and secretory calcifications in the breast and Paget’s disease of the nipple.

**Figure 2:**
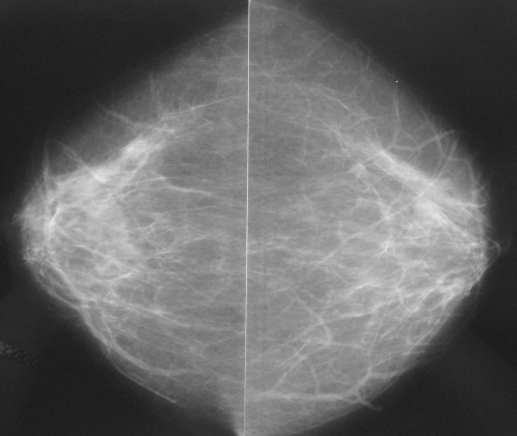
A 68-year-old woman, presented with scaling of the right nipple. Bilateral CC mammograms show scattered fibrograndular densities with no definite abnormality. Additional US (not shown) showed no abnormality. She had wide local excision and showed only Paget’s disease of the nipple. She was well up to 3-years follow-up.

**Figure 3:**
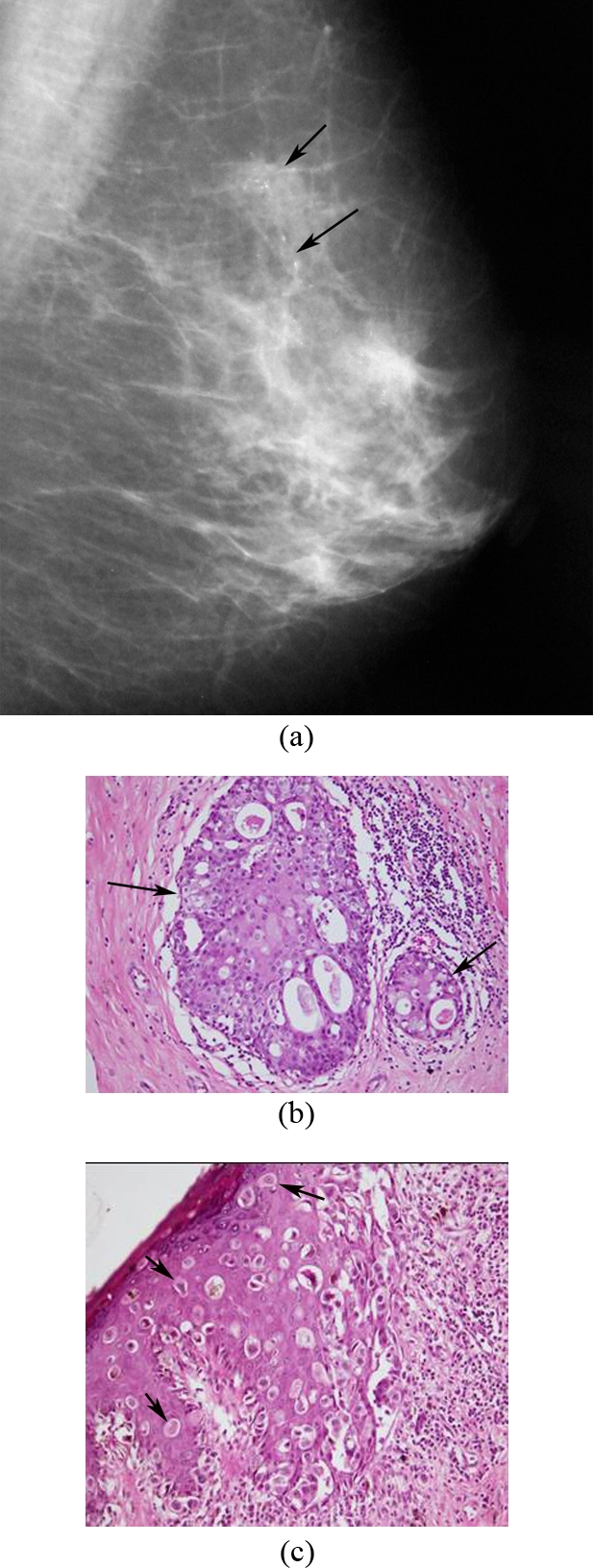
A 65-year-old woman, presented with scaling and erythema of the left nipple; (a) Left MLO mammogram shows segmental distribution of pleomorphic microcalcifications (arrows) in the upper quadrant. Simple mastectomy revealed DCIS in the breast and Paget’s disease of the nipple; (b) Photomicrograph shows slightly dilated duct (arrows) containing malignant epithelial cells with cribriform arrangement (H&E, x200); (c) Photomicrograph shows infiltration of malignant cells called Paget cells (arrows) among squamous cells of epidermis. These malignant cells are large and show pleomorphism containing hyperchromatic nuclei (H&E, x200).

**Figure 4:**
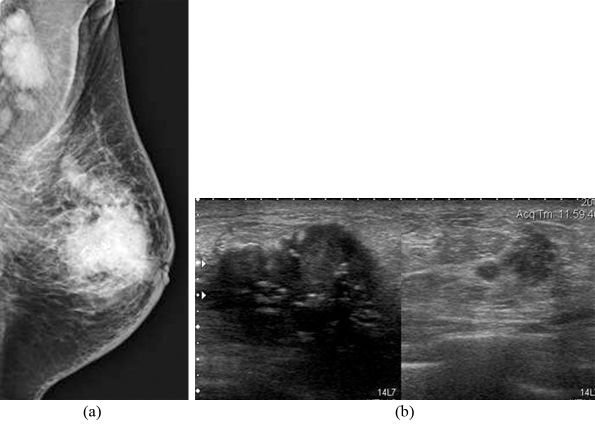
A 60-year-old woman, presented with a palpable left breast mass; (a) Left MLO mammogram shows heterogeneously dense breast with a large ill-defined mass containing microcalcifications with mild thickening of the overlying skin, nipple retraction, and multiple enlarged axillary nodes; (b) Composite US images of the left breast show two irregular masses with internal calcifications in the big mass. Modified radical mastectomy revealed multifocal invasive ductal carcinoma with extensive intraductal component, axillary node metastases, and Paget’s disease of the nipple.

**Figure 5:**
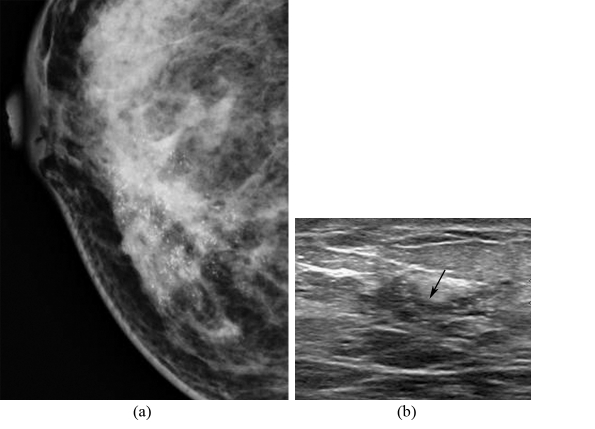
A 43-year-old woman, presented with right bloody nipple discharge; (a) Right CC mammogram shows heterogeneously dense breast and segmental distribution of pleomorphic microcalcifications in the inner quadrant; (b) US of the right inner quadrant shows ductal dilatation (arrow) filled with echogenic content and microcalcifications. Core biopsy under US guidance showed DCIS with microinvasion. Modified radical mastectomy revealed DCIS with microinvasion and Paget’s disease of the nipple.

**Figure 6:**
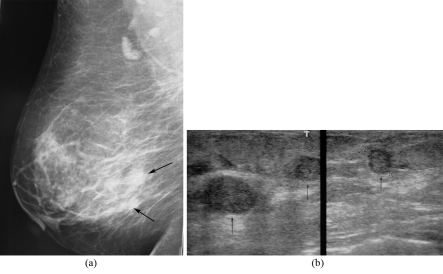
Multifocality. A 58-year-old woman, presented with a palpable right breast mass and erosion of the right nipple with serous discharge; a) Right MLO mammogram shows scattered fibroglandular densities with two ill-defined masses (arrows); (b) Composite US images of the right breast show three hypoechoic breast masses (arrows). Modified radical mastectomy revealed multifocal invasive ductal carcinomas and Paget’s disease of the nipple.

**Figure 7:**
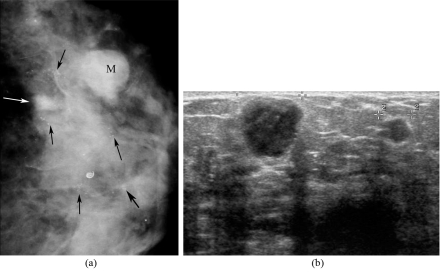
Multicentricity. A 54-year-old woman, presented with a palpable left breast mass with erosion and bloody nipple discharge; (a) Left CC mammogram shows heterogeneously dense breast, a small ill-defined mass (white arrow) and a round shaped, obscured border mass (M) in the outer quadrant, and multiple groups of pleomorphic microcalcifications (black arrows); (b) US image of the left breast shows two hypoechoic masses. Modified radical mastectomy revealed multicentric DCIS with microinvasion and invasive ductal carcinoma with extensive intraductal component with Paget’s disease of the nipple.

The prevalence of Paget’s disease of the breast was 1.14% of all breast carcinomas at the authors’ institution. Of the 27 patients with Paget’s disease, only 16 had complete clinical and imaging studies done, and this group constituted the basis of this study. All 16 patients were women, ages ranging from 36–68 years (mean age, 50.31 years). Eleven patients presented with clinical findings suggestive of Paget’s disease of the breast (Figure 1). Seven of these 11 patients also had associated palpable mass(es). Four patients presented with a palpable mass alone and one presented with bloody nipple discharge alone. Mammography was performed in all 16 patients but US was performed in 15. Of the 16 mammographic studies, two were negative. Of the 15 US studies, three were negative. Of these three negative US studies, two also had negative mammography (Figure 2) and one had pleomorphic microcalcifications on mammogram (Figure 3). US was helpful in detecting multifocality in two patients who had solitary mass on mammogram (Figure 4), and guiding biopsy in two patients with nonpalpable microcalcifications seen from mammograms (Figure 5). Altogether, there were seven patients who had multifocality or multicentricity (Figures 6 and 7). None had bilateral breast carcinomas. The breast parenchymal pattern was scattered fibroglandular density in six (37.5%), heterogeneously dense in eight (50%) and extremely dense in two (12.5%). Modified radical mastectomy was performed in 13 patients, simple mastectomy in two, and wide local excision in one patient. Pathological findings of the breasts were ductal carcinoma in situ (DCIS) (*n* = 3), invasive ductal carcinoma (IDC) (*n* = 10), metaplastic carcinoma (*n* = 1), invasive lobular carcinoma (ILC) (*n* = 1), and Paget’s disease of the nipple without underlying breast carcinoma (*n* = 1).

Summarised clinical presentation, mammographic, US and pathological findings are shown in Table 1.

**Table 1:** Clinical presentation, mammographic, US findings and pathological findings in 16 patients with Paget’s disease of the breast.

**Clinical presentation**	**Mammographic findings**							**Ultrasonographic findings**		**Pathological findings**
	**Negative**	**Mass**	**Nipple-areolar change**	**Micro calcification**	**Asymmetrical density**	**Skin thickening**	**Axillary node**	**Mass**	**Micro calcification**	
Nipple changes suggestive of Paget (*n* = 11)										
Alone (*n =* 4)	2			2					1	DCIS (2) IDC (1) No underlying CA (1)
With mass (n = 7)		6	1	3	3		2	7	3	DCIS (1) IDC (4) ILC (1) Metaplasic CA (1)
Palpable mass (*n* = 4)		3	2	4	2	2	2	3*	2	IDC (4)
Bloody nipple discharge (*n* = 1)				1					1	DCIS with microinvasion

* US performed in 3; CA = carcinoma

## DISCUSSION

Paget’s disease of the breast accounts for 1–4.3% of all the breast cancers [2,3]. The authors found a prevalence of 1.14% in their institution, which is not different from other studies. The mean age of the patients in this study was 50.31 years, which is similar to the study by Günhan-Bilgen *et al.* [3] but makes them younger than patients in other studies [5,8,9]. Most patients with Paget's disease of the breast present with nipple areolar changes either with or without associated palpable mass in the breast. Occasionally, patients may present with a palpable mass only and Paget’s disease of the breast is found as incidental histological finding in the specimen [2–5,10]. In this study, most patients (11/16) presented with nipple areolar change suggestive of Paget’s disease and only four presented with mass without nipple-areolar change. However, mammography found nipple retraction in two of these patients. These findings showed that the patients neglected their nipple changes, even though they had breast mass.

Paget’s disease of the breast is nearly always a sign of underlying breast malignancy with prevalence of associated cancer range from 67–100% [11,12]. The associated carcinoma can be either carcinoma in situ or invasive cancer [3,5,11,13]. Patients with typical clinical presentations suggestive of Paget’s disease would have more early breast carcinoma and less extension of disease than those patients without clinical presentations suggestive of Paget’s disease but with pathological findings showing Paget cells [3,5]. Almost all patients (15/16) had underlying breast malignancy and 75% (12/16) were invasive carcinoma. When compared with other studies [3,5,11], the patients in this study had more invasive carcinoma, which may reflect the late presentation of the patients. The incidence of axillary lymph node involvement in Paget’s disease of the breast has been reported to be 50–65% in patients with a palpable mass and 0–15% in patients without a palpable mass [10,12]. Axillary lymph node involvement was detected in 36.4% of the patients in this study who presented with a palpable mass and none in patients who had no palpable mass.

Because Paget’s disease of the breast is always associated with underlying breast carcinoma, mammography may help clinicians to find the underlying malignancy. The sensitivity of the mammography seems to be significantly higher in patients with palpable masses compared to patients with disease confined to the nipple and do not have palpable masses [5,11]. All the 11 patients in this study who had palpable breast mass(es) and one who had bloody nipple discharge had positive mammography. Two out of four patients who had no palpable mass had positive mammography. The reported mammographic findings include skin, nipple and areolar thickening, nipple retraction, mass(es), microcalcifications, asymmetrical density, and architectural distortion [2–6]. Masses and microcalcifications were the most common mammographic findings in this study. The rate of negative mammography was low (2/16 = 12.5%) in this study compared to other studies [2,3,5,11]. This may be the result of the relatively advanced presentation of the patients. Additional US was reported to increase the sensitivity of mammography in the detection of carcinoma especially in patients with negative mammography [3,14]. However, US did not improve cancer detection in this study. The two patients who had negative mammography also had negative US but US helped to detect multifocality in two patients and guide biopsy in nonpalpable lesions. To the authors’ knowledge, Günhan-Bilgen *et al.* [3] described the first report of US features in patients with Paget’s disease of the breast. The US findings in this study were not different from that of their study. Approximately 42–63% of underlying carcinoma in Paget’s disease have been reported to be multifocality [11,15]. In this study, a multifocality of 43.7% (7/16) was found. Since mammography and US may not demonstrate abnormality in the breast even clinically suggestive of Paget’s disease of the breast, magnetic resonance imaging (MRI) has been introduced to depict the lesions especially in a patient without palpable mass [3,16,17]. MRI was not performed in both patients who had negative mammography and US. One of these two patients had wide local excision and the patient was still free of underlying breast carcinoma after follow-up for 3 years. The other patient had simple mastectomy and found ductal carcinoma in situ close to the nipple.

Mastectomy with or without axillary node dissection was established as standard treatment [10,11] for Paget’s disease of the breast because of the high association of underlying breast carcinoma. Currently, breast-conserving therapy has been adopted. Patients must be selected carefully depending on clinical and mammographic findings [10,18]. In this study, only one patient had breast-conserving therapy because most of the patients had advanced lesions.

## CONCLUSION

Patients with Paget’s disease of the breast have a high incidence of an underlying breast carcinoma. Most of the patients in this study presented late and had more positive mammogram as compared to other studies. Mammography should be performed to identify the underlying breast carcinoma. Those who have only nipple areolar changes without a palpable mass have less positive mammography and less invasive carcinoma.
